# Patient Attributes Associated With Better Long-Term Outcomes on Palliative Milrinone

**DOI:** 10.7759/cureus.7979

**Published:** 2020-05-05

**Authors:** Toishi Sharma, Kathir Balakumaran, Varun Tandon, Nihar Shah, Sabeena Arora

**Affiliations:** 1 Internal Medicine, Hartford Hospital, Hartford, USA; 2 Cardiology, Cleveland Clinic Ohio, Cleveland, USA; 3 Internal Medicine, University of Arizona College of Medicine, Phoenix, USA; 4 Internal Medicine, University of Connecticut Health, Hartford, USA; 5 Cardiology, Hartford Hospital, Hartford, USA

**Keywords:** milrinone, long-term outcome, survival, inotrope, prognostic factors, phosphodiesterase inhibitor 3, patient characteristics, comorbidities, heart failure, palliative milrinone

## Abstract

Milrinone is a phosphodiesterase three inhibitor used as an inotrope in patients with advanced heart failure with reduced ejection fraction (HFrEF). Its action is independent of β-receptor stimulation, which makes it preferable in patients who are on β blockers as part of a guideline-directed neurohormonal blockade.

There have been numerous studies evaluating the risks, benefits, and mortality associated with milrinone in the management of chronic heart failure patients. Time and again, there has been concern regarding the undesirable outcomes associated with it, including higher mortality and cardiac arrhythmias. Additionally, it has been difficult to determine whether milrinone or disease progression is responsible for adverse outcomes and mortality. In light of such discrepancy, the selection of patients for milrinone remains challenging. We hypothesized that there are underlying patient characteristics that influence the response to milrinone and may predict milrinone's adverse outcomes in spite of milrinone. A retrospective study review of 10 patients on palliative milrinone was conducted to identify these factors with a mean follow-up of 36 months.

During the study period, four of 10 patients died. These four patients were on milrinone for a mean of 11.5 months. The attributes of the survivors compared to the deceased included lower age at start of therapy (67.5 vs 79 y), female gender (66% vs 33%), non-ischemic cardiomyopathy (33% vs 50%), associated diagnosis of atrial fibrillation/flutter(50% vs 25%), hyperlipidemia (66% vs 50%), or anemia (83% vs 75%), presence of chronic resynchronization therapy (CRT) (66% vs 25%), and implantable cardioverter-defibrillator (ICD) (16% vs 0%), as well as lower sodium (136 vs 140 mEq), chloride (101.5 vs 104.5 mEq), potassium (4.07 vs 4.23 mEq), and creatinine (1.3 vs 1.8 mg/dL)

Conversely, the deceased patients were more likely to have coronary artery disease (75% vs 33%), diabetes mellitus (50% vs 16%), hypertension (100% vs 83%), chronic kidney disease (75% vs 66%), peripheral vascular disease (25% vs zero), higher pulmonary artery pressures (54 vs 50.5%), and history of percutaneous coronary intervention (PCI) or coronary artery bypass graft (CABG) (50% vs 16%). These trends exhibit patient characteristics that may predict better outcomes on long-term milrinone although larger studies are needed to assess the statistical significance of these findings.

## Introduction

Milrinone is a phosphodiesterase inhibitor used as a positive inotropic agent in patients with advanced heart failure and cardiogenic shock. It acts by increasing intracellular cyclic adenosine monophosphate (cAMP), thereby increasing the concentration of intracellular calcium and leading to a positive inotropic effect, independent of β-receptor stimulation in the myocardial cells, making it different from dobutamine and dopamine and preferable in heart failure patients who are on β blockers as part of a guideline-directed neurohormonal blockade. Milrinone also reduces the left ventricular filling pressure in chronic heart failure patients and helps reduce pulmonary artery pressure by vasodilating pulmonary vasculature via cAMP, thus improving right ventricular function [1].

Although originally used in the hospital setting, intravenous infusions of milrinone are now being increasingly used on an outpatient basis [2]. It is being used primarily as a bridge to recovery from an acute hemodynamically compromised state and in advanced-stage heart failure patients awaiting advanced heart failure therapies such as mechanical circulatory support and heart transplant. Additionally, it is used in stage D heart failure patients who are not candidates for advanced heart failure therapies as palliative therapy for symptom improvement [3].

Multiple studies that evaluated the risks, benefits, and mortality associated with milrinone in the management of chronic heart failure patients saw undesirable outcomes, including higher mortality and cardiac arrhythmias. Also, it has been difficult to determine whether milrinone or disease progression are responsible for these adverse outcomes. Despite the considerably homogeneous prevalence of milrinone’s adverse effects, the association of their development with the duration of treatment remains debatable and although studies have largely confirmed increased mortality, there are emerging collateral smaller studies that have reported the relative safety of long-term milrinone, the longest being up to 10 months.

Owing to this lack of knowledge, the selection of patients with advanced heart failure with reduced ejection fraction (HFrEF) who are more likely to respond to milrinone with fewer side effects and improved survival remains challenging. We sought to study patient characteristics that influence the response to milrinone and predict milrinone-related side effects.

## Materials and methods

This is a single-center retrospective study conducted at Trinity Health St Francis Hospital with a mean follow-up of 36 months on long-term milrinone. A manual chart review of electronic health records of 10 patients was conducted. The primary endpoint of the study was overall survival on long-term milrinone. Patient-related attributes were studied between the survivors and deceased to identify the characteristics associated with better (survivors) or poorer (deceased) outcomes.

## Results

Over the follow-up period of 36 months, a total of four patients died. The mean duration of milrinone therapy for the deceased group was 11.5 months. The characteristics of the patients who survived at the end of the study period compared to the deceased were as follows: lower age at start of therapy (67.5 vs 79 years), female gender (66% vs 33%), non-ischemic cardiomyopathy (33% vs 50%), associated diagnosis of atrial fibrillation/flutter (50% vs 25%), hyperlipidemia (66% vs 50%), or anemia (83% vs 75%), presence of chronic resynchronization therapy (CRT) (66% vs 25%) and implantable cardioverter-defibrillator (ICD) (16% vs zero), as well as lower sodium (136 vs 140 mEq), chloride (101.5 vs 104.5 mEq), potassium (4.07 vs 4.23 mEq), and creatinine (1.3 vs 1.8 mg/dL) levels.

Conversely, the deceased patients were more likely to have a history of coronary artery disease (75% vs 33%), type 2 diabetes mellitus (50% vs 16%), hypertension (100% vs 83%), chronic kidney disease (75% vs 66%), peripheral vascular disease (25% vs zero), higher pulmonary artery pressures (54% vs 50.5%), and a history of percutaneous coronary intervention (PCI) or coronary artery bypass grafting (CABG) (50% vs 16%) (Table 1).

**Table 1:** Factors associated with survival on long-term milrinone therapy mEq: milliequivalent

Patient Characteristics	Deceased	Survivors
Mean age at the start of therapy	79 years	67.5 years
Proportion of females	33%	66%
Non-ischemic cardiomyopathy	33%	50%
Hyperlipidemia	50%	66%
Atrial fibrillation/flutter	25%	50%
Presence of chronic resynchronization therapy	25%	66%
Sodium level	140 mEq	136 mEq
Chloride level	104.5 mEq	101.5 mEq
Coronary artery disease	75%	33%
Chronic kidney disease	75%	33%
History of percutaneous coronary intervention or coronary artery bypass graft	50%	16%
Peripheral vascular disease	25%	0%
Diabetes mellitus	50%	16%
Hypertension	100%	83%
Pulmonary artery pressure	54 mmHg	50.5 mmHg

## Discussion

According to Hashim et al, survival on inotropes for patients who are not candidates for transplant/left ventricular assist device (LVAD) is modestly better than previously reported but remains poor [4]. A meta-analysis of 21 randomized trials showed that phosphodiesterase inhibitor (PDI)-like milrinone are associated with significantly higher mortality and cardiac arrhythmias when compared to placebo irrespective of the concomitant use of other vasodilating agents, the severity of heart failure, and the derivative or molecule of PDI used [5]. Previous trials, a meta-analysis, and several reviews have supported similar deleterious effects with PDIs [5-6].

It is remarkable to note that despite the considerably homogeneous prevalence of these adverse effects, the association of their development with the duration of treatment remains debatable. For a shorter duration of treatment, ranging from days to weeks, studies found increased mortality and arrhythmias associated even after hours of milrinone infusion while others demonstrated safety, efficacy, and tolerability over periods of up to eight weeks [7-11].

Although studies have largely confirmed increased mortality, there are emerging collateral smaller studies that have reported the relative safety of long-term milrinone [12-14]. One such prospective study evaluating survival-to-transplant in patients on milrinone followed patients over a mean period of 160 days [15]. Similarly, Andres et al. demonstrated reduced hospitalization, days spent in a hospital, and emergency department (ED) visits in 32 patients over a mean period of observation of 294 days [2].

It is important to study if patient-specific factors like age, gender, and co-morbid conditions may be associated with safety, efficacy, and event-free duration of treatment. The fact that studies with encouraging long-term outcomes excluded patients with certain characteristics (e.g., infection, acute renal failure, elevated transaminases, and so on) further raised curiosity to identify patient-related factors that could positively or negatively influence a milrinone associated adverse drug profile [2,15].

Similar to our results, Lee et al. found the female gender and the presence of ICD to be associated with better outcomes on milrinone [16]. On the contrary, Harhash et al. found a lower rate of atrial fibrillation in patients on milrinone [17]. As we continue to explore various factors that may help predict better candidature for milrinone, recent studies have also shed light on the favorable effects of extended-release oral milrinone in improving end-stage heart failure symptoms [18]. This approach may significantly expand the utilization of milrinone in the future.

This study shows that underlying patient characteristics can influence the response to milrinone and sets the stage for larger studies to pilot the way for the evidence-based selection of candidates in the future.

## Conclusions

Factors associated with improved survival on long-term milrinone included younger age at the start of therapy, female gender, non- ischemic cardiomyopathy, atrial fibrillation/flutter, hyperlipidemia, anemia, and the presence of CRT and ICD. Similarly, lower sodium, chloride, potassium, and creatinine levels were linked to better outcomes. Conversely, coronary artery disease, diabetes mellitus, hypertension, chronic kidney disease, peripheral vascular disease, higher pulmonary artery pressures, and a history of PCI or CABG were associated with higher mortality in patients on milrinone. These trends exhibit potential patient characteristics that may predict better outcomes on long-term milrinone.
